# Multi analyte profiling and variability of inflammatory markers in blood and induced sputum in patients with stable COPD

**DOI:** 10.1186/1465-9921-11-41

**Published:** 2010-04-22

**Authors:** Shawn D Aaron, Katherine L Vandemheen, Timothy Ramsay, Chun Zhang, Zafrira Avnur, Tania Nikolcheva, Anthony Quinn

**Affiliations:** 1 Department of Medicine, The Ottawa Health Research Institute, University of Ottawa, 501 Smyth Road, Ottawa, ON, K1H 8L6, Canada; 2 Roche Pharmaceuticals CRED, Roche Palo Alto, 3431 Hillview Ave, A2-246 Palo Alto, CA 94304 USA

## Abstract

**Background:**

We analyzed serial concentrations of multiple inflammatory mediators from serum and induced sputum obtained from patients with stable COPD and controls. The objective was to determine which proteins could be used as reliable biomarkers to assess COPD disease state and severity.

**Methods:**

Forty-two subjects; 21 with stable COPD and 21 controls, were studied every 2 weeks over a 6-week period. Serum and induced sputum were obtained at each of 3 visits and concentrations of 19 serum and 22 sputum proteins were serially assessed using multiplex immunoassays. We used linear mixed effects models to test the distribution of proteins for an association with COPD and disease severity. Measures of within- and between-subject coefficients of variation were calculated for each of the proteins to assess reliability of measurement.

**Results:**

There was significant variability in concentrations of all inflammatory proteins over time, and variability was greater for sputum proteins (median intra-subject coefficient of variation 0.58) compared to proteins measured in serum (median intra-subject coefficient of variation 0.32, P = 0.03). Of 19 serum proteins and 22 sputum proteins tested, only serum CRP, myeloperoxidase and VEGF and sputum IL-6, IL-8, TIMP-1, and VEGF showed acceptable intra and inter-patient reliability and were significantly associated with COPD, the severity of lung function impairment, and dyspnea.

**Conclusions:**

Levels of many serum and sputum biomarkers cannot be reliably ascertained based on single measurements. Multiple measurements over time can give a more reliable and precise estimate of the inflammatory burden in clinically stable COPD patients.

## Introduction

There is increasing evidence that chronic obstructive pulmonary disease (COPD) is associated with chronic systemic inflammation as well as inflammation in the airways and lung parenchyma [1,2]. Previous studies have used quantitative enzyme-linked sandwich immunoassays (ELISA) to measure a limited number of biomarker proteins and have shown that serum C-reactive protein levels (CRP), and sputum neutrophil chemo-attractants such as interleukin-8 (IL-8), and tumor necrosis factor-α (TNFα), are significantly higher in patients with COPD compared to controls [3,4]. Newer assay methods, which use multiplex sandwich ELISA technology, allow measurement of up to 30 cytokines using a single blood sample, and are thus potentially more useful[5].

A recent meta-analysis of 652 studies showed that only sputum neutrophils, IL-8, TNFα, and CRP were able to distinguish between differing stages of COPD disease severity [6]. However in many of the studies reviewed in the meta-analysis there is evidence of variability in the levels of individual inflammatory mediators between subjects. The significance of this heterogeneity is difficult to ascertain as most studies have focused on identifying differences in mediators between COPD patients and controls and have used samples collected at a single time point. Thus it is unclear whether levels of inflammatory mediators vary widely within stable COPD patients over time. Without knowledge of the inherent variability of the factor being analyzed, it is difficult to know whether the protein, measured in blood and sputum, can be used as a reliable biomarker to assess disease state and disease severity.

Our study had two major objectives. The first objective was to use multi-analyte profiling of numerous inflammatory proteins in induced sputum and serum to determine which proteins could be used as reliable biomarkers to assess COPD disease state and disease severity. The second objective was to describe the intra- and inter-patient variability in inflammatory proteins in induced sputum and serum in patients with stable COPD compared to controls.

## Methods

### Study subjects

We recruited 4 categories of subjects aged 35 years or older into this study; 1) Current smokers with COPD, 2) Former smokers with COPD, 3) Current smokers with no evidence of COPD or airflow obstruction, and 4) Lifetime non-smokers with no evidence of COPD or airflow obstruction. Groups 3 and 4 were control groups. This design allowed us to assess for current smoking as an independent risk for altered inflammatory cytokine profiles, and allowed us to assess potential interactions of smoking and disease state. All patients with COPD had to have at least GOLD stage II-IV COPD and a 10 pack-year history of smoking. We excluded patients with a history of COPD exacerbation within one month prior to study entry, asthma or atopy, patients using chronic oral prednisone, those not able to perform spirometry, and those subjects with other conditions which may cause inflammation, including; current cancer, infections, rheumatoid arthritis, lupus, scleroderma, or a recent myocardial infarction within 3 months. The study was approved by The Research Ethics Board of The Ottawa Hospital and all subjects signed written, informed consent.

### Study design

This was a prospective cohort study. Subjects were seen on 3 occasions at 2-week intervals over a 6 week period. On each visit, patients underwent assessments of measurements of dyspnea intensity using the Baseline Dyspnea Index [7] and measurements of respiratory symptoms (including dyspnea, cough, sputum production) using the American Thoracic Society Questionnaire [8]. Spirometry was performed at each visit along with a clinical review to ensure clinical stability.

### Sample collection

Induced sputum and blood samples were collected at each visit. Sputum induction was performed according to previously described protocols [9]. Subjects inhaled 5 mls of 0.9% saline, 3% saline and 5% saline successively via an aerosol nebulizer (Flaem Aerosol Universal III) at maximal output (0.8 ml/min) for five minutes until 2 ml of sputum was obtained. Sputum quality was assessed using Gram staining, samples containing >25 squamous epithelial cells per low power field were discarded. Sputum was diluted with an equal amount of 0.1% dithiothreitol in Hanks solution and incubated on a rocking platform for 15 min at room temperature to digest mucus. The suspended mixture was allowed to stand at room temperature for 10 minutes and the sputum digests were centrifuged at 275 × *g *to sediment cellular constituents. Cell supernatants and centrifuged serum were collected and stored frozen at -70°C. Samples were batched and shipped on dry ice at the halfway point of the study and at the study termination. Samples were kept frozen from 1 day up to 3 months. Frozen samples were immediately thawed on receipt at the laboratory and processed for assessment of protein levels.

### Measurement of analytes

Serum and sputum levels of inflammatory mediators were measured using two multiplex platforms: Luminex multi-analyte profiling performed at Rules Based Medicine (Austin, TX) [5,10] and SearchLight, performed at Pierce Biotechnology (Woburn, MA) [11]. Two complementary multiplex assays were used since neither platform could assay for all of the biomarkers that were measured in this study. Proteins chosen for analysis were those that were thought to have known or potential significance in the pathobiology of COPD. Twenty-eight proteins were assayed for in serum on the subject's first visit, but 9 proteins (α2-macroglobulin, IL-1β, IL-RA, IL-15, MMP-2, MMP-9, IL-10, and TNFα) were not measured serially on all three visits because of serum concentrations below the limit of detection of the assays, or because there was no significant difference on visit 1 between serum levels in COPD patients and in controls. Twenty-two proteins were serially assayed from sputum. TNFα and Eotaxin-1 could not be measured in sputum, because dithiothreitol used for sputum processing interfered with measurements of these cytokines. The total protein concentration of each individual sputum sample was determined and sputum biomarker levels were reported normalized for the sputum total protein concentration. Total protein was measured in sputum using the Coomassie Plus Protein assay. The reagent was added to the sputum supernatant and absorbance was read at 595 nm.

### Data analysis

We tested the distribution of biomarkers for an association with COPD using a linear mixed effects model including disease (COPD or control), smoking, and the interaction between disease and smoking as fixed effects, and visit number within each subject as a random effect for each protein. A log transformation was used for those analytes whose concentration distributions were highly skewed. A second linear mixed effects model was also constructed adding age and gender into the original model as covariates to adjust for potential effects of these variables. We assessed associations between individual protein levels and lung function and dyspnea using a linear mixed effect model adjusting for age and gender. In these models lung function variables and dyspnea scores were fixed effects and patient visit number (1, 2 or 3) was modeled as a random effect.

The analytical variation of the measurements was obtained from the assay manufacturers. Within-subject coefficient of variation (WCV) and the between-subject coefficient of variation (BCV) were calculated for each of the proteins to assess for the intra- and inter-patient variability respectively in the levels of the inflammatory proteins. The reference change value for each analyte measurements was calculated from the analytic variation and the within subject coefficient of variation using the Harris formula [12]. Bootstrap re-sampling was performed to estimate the 90% confidence intervals of the within- and between-subject coefficients of variation.

## Results

### Subject Characteristics

We studied 42 subjects prospectively over a 6-week period. Twenty-one subjects had COPD; 8 were active smokers, and 13 were former smokers. Twenty-one controls without COPD were studied; 9 were active smokers and 12 had never smoked. Patient characteristics of the 4 groups of patients are listed in Table 1, along with their baseline lung function data. The mean post-bronchodilator FEV1 (after inhalation of 200 ug of salbutamol) was 46% and 44% predicted in the non-smoking and smoking cohorts with COPD respectively. Lung function was normal in the control group.

**Table 1:** Baseline Characteristics of the Study Subjects:

	COPD non-smokers (N = 13)	COPD smokers (N = 8)	Control non-smokers (N = 12)	Control smokers (N = 9)
**Mean age (95% CI)**	72.8 (69.4-76.3)	62.3 (58.7 - 65.8)	66.7 (62.4-70.9)	56.4 (52.7-60.1)

**Female Gender**	7 (54%)	5 (63%)	7 (58%)	6 (67%)

**Mean BMI (95% CI)**	28.3 (25.4-31.2)	25.5 (20.9-30.2)	25.8 (23.9-27.7)	26.0 (24.5-27.6)

**Pack Years (95% CI)**	44.5 (33.7-55.2)	53.8 (44.5-63.0)	0	29.3 (22.0-36.7)

**Post-bronchodilator FEV1% predicted**	46 (39 - 53)	44 (33 - 54)	101 (92-111)	102 (93-111)

**GOLD Stage II**	6 (46%)	3 (38%)	0	0
**GOLD Stage III**	6 (46%)	3 (38%)	0	0
**GOLD Stage IV**	1 (8%)	2 (25%)	0	0

**History of Chronic Bronchitis**	7 (54%)	8 (100%)	0 (0%)	3 (33%)

**Total lung capacity % predicted**	122 (108 - 136)	117 (106-127)	103 (96-110)	106 (99-114)

**Residual volume % predicted**	172 (132 - 211)	176 (150-201)	93 (81-104)	94 (81-108)

**Diffusion capacity (DLCO % predicted**	64 (56 - 73)	64 (50-78)	86 (82-90)	78 (71-84)

**Baseline Dyspnea Index Score (95% CI)**	6.0 (4.7 - 7.4)	6.5 (4.7 - 8.2)	11.6 (11.2-11.9)	10.9 (10.1-11.6)

**Use of inhaled corticosteroids**	10 (77%)	7 (88%)	0	0

### Serum Biomarker Results

Table 2 shows the mean serum concentrations for the biomarkers averaged over the 3 patient visits. A linear mixed effects model was fitted for each biomarker. Of the 19 serum proteins that were serially assayed, four (21%) were found to be significantly associated with COPD. Serum levels of Eotaxin2, C reactive protein (CRP), myeloperoxidase and vascular endothelial growth factor (VEGF) were significantly higher in patients with COPD compared to controls. Smoking did not exert a confounding effect; these four serum proteins were elevated in both smokers and non-smokers with COPD compared to respective controls. Similarly, adjustment for age and gender did not alter the association between the four serum markers and presence of COPD. Serum IL-6 and IL-8 could not be completely assessed since their values were below the limits of detection of the assay for some COPD patients and for many of the controls.

**Table 2:** Mean serum protein concentrations averaged over the 3 patient visits:

Serum Protein	COPD non-smokers (N = 13)	COPD smokers (N = 8)	Control non-smokers (N = 12)	Control smokers (N = 9)	P value COPD vs controls
**Eotaxin2 (pg/ml)**^**#**^	554.2 (352.5)	465.6 (327.4)	364.3 (158.3)	475.5 (766.1)	0.025

**IP-10 (pg/ml)**^**#**^	170.2 (95.2)	164.8 (126.4)	125.8 (26.4)	100.7 (44.3)	0.22

**TNF-R1 (pg/ml)**^**#**^	1489.4 (303.0)	1312.1 (257.3)	1430.9 (268.9)	1442.7 (268.0)	0.67

**TNF-RII (pg/ml)**^**#**^	194.0 (118.8)	268.4 (143.8)	241.9 (116.2)	235.9 (33.9)	0.10

**TIMP-2 (pg/ml)**^**#**^	25028.7 (5273.1)	27639.6 (4570.7)	30286.8 (4946.8)	26514.6 (6425.1)	0.046

**HGF (pg/ml)**^**#**^	21112.5 (12544.1)	19937.9 (10785.1)	24076.2 (14625.4)	25125.4 (9975.2)	0.56

**KGF (pg/ml)**^**#**^	82.1 (34.5)	56.9 (26.0)	65.3 (20.8)	66.1 (15.7)	0.94

**MMP-13 (pg/ml)**^**#**^	1768.8 (2561.6)	718.9 (450.6)	774.5 (421.6)	733.6 (328.7)	0.58

**GROα (pg/ml)**^**#**^	123.3 (61.4)	93.4 (33.6)	114.8 (53.0)	113.6 (65.9)	0.21

CRP (ug/ml)*	13.6 (11.4)	7.8 (9.1)	4.1 (3.2)	4.4 (4.0)	0.042

ENA-78 (ng/ml)*	2.3 (2.69)	1.8 (0.6)	2.5 (1.7)	2.9 (1.9)	0.13

IL-13(pg/ml)*	37.5 (7.0)	46.8 (13.4)	40.4 (10.3)	37.9 (7.9)	0.17

IL-18 (pg/ml)*	302.1 (165.4)	329.0 (106.7)	281.9 (170.9)	256.3 (109.1)	0.17

IL-6 (pg/ml)*	8.0 (7.1)	6.4 (5.1)	2.3 (0.7)	Below detection limits	0.20

IL-8 (pg/ml)*	15.1 (12.1)	11.4 (3.7)	11.1 (3.4)	11.5 (3.9)	0.30

MCP-1 (pg/ml)*	279.1 (181.5)	259.7 (101.1)	182.3 (35.6)	198.7 (42.4)	0.16

Myeloperoxidase (ng/ml)*	523.7 (546.2)	597.9 (298.9)	379.6 (308.8)	430.3 (248.9)	0.031

TIMP-1 (ng/ml)*	162.7 (33.1)	160.7 (21.2)	162.3 (31.5)	146.0 (33.2)	0.61

VEGF (pg/ml)*	753.1 (217.9)	999.5 (826.2)	586.2 (192.1)	674.5 (149.4)	0.035

Values in brackets are the standard deviations of the mean.

P values were assessed using linear mixed effects models adjusted for age and gender (see methods section for details).

# = Measured using SearchLight multiplex sandwich ELISA

* = Measured using Luminex bead- based suspension array assays

IP-10 = interferon-inducible protein 10, TNF-R1 = tumour necrosis factor receptor I, TNF-RII = tumour necrosis factor receptor 2, TIMP-2 = tissue inhibitors of metalloproteinases 2, HGF = hepatocyte growth factor, KGF = keratinocyte growth factor, MMP-13 = matrix metalloproteinase 13, GROα = growth-related oncogene alpha, CRP = C-reactive protein, ENA-78 = epithelial neutrophil-activating peptide 78, IL = interleukin, MCP-1 = monocyte chemotactic protein 1, TIMP-1 = tissue inhibitors of metalloproteinases 1, VEGF = vascular endothelial growth factor.

Table 3 shows values for within-subject mean coefficients of variation, between-subject coefficients of variation, analytical variation and reference change values for the 19 serum inflammatory proteins in the COPD cohort. The median within-subject coefficient of variability for serum proteins was 32%. A minimally acceptable variation in serum concentrations for each subject (defined as a within subject CV < 60%) was seen for most proteins with the exception of Eotaxin2, HGF, and MMP13 which had within-subject CV values exceeding 60%. Between-subject variation was also assessed, and results suggest that MMP13 and ENA 78 had high inter-subject variability (>100%) in the concentrations of these proteins between COPD patients. Serum levels of C reactive protein (CRP), myeloperoxidase and VEGF, but not Eotaxin2, showed acceptable intra- and inter-subject variability. Reference change values for most proteins (Table 3) were relatively high (median reference change value = 91%, range 48% to 246%) suggesting that for most proteins individual measurements in a given patient need to change by >48% from baseline before a statistically significant change in the measurement can be assumed.

In the patients with COPD serum myeloperoxidase and VEGF were significantly and negatively associated with the FEV_1 _(Table 4). Serum CRP, myeloperoxidase and VEGF all showed significant associations with patient dyspnea and were negatively associated with diffusion capacity.

**Table 3:** Within-subject and between-subject coefficients of variation, analytical variation and reference change value in COPD serum samples:

Serum Protein	Within-subject CV (%) (90% CI)	Between-subject CV (%) (90% CI)	Analytical variation (%)	Reference change value (%)
**Eotaxin2**	64 (55 - 77)	65 (50 - 77)	12.1	180.42

**IP-10**	49 (44 - 57)	62 (50 - 67)	12.2	139.87

**TNF-R1**	26 (20 - 33)	20 (13 - 29)	8.8	76.03

**TNF-RII**	50 (42 - 59)	59 (40 - 68)	8.6	140.53

**TIMP-2**	21 (17 - 26)	20 (13 - 24)	8.4	62.65

**HGF**	71 (59 - 84)	56 (37 - 65)	14.3	200.62

**KGF**	55 (46 - 62)	46 (35 - 57)	8.3	154.08

**MMP-13**	88 (74 - 103)	151 (57 - 172)	11.0	245.66

**GROα**	32 (26 - 37)	48 (28 - 56)	8.2	91.50

**CRP**	53 (40 - 69)	94 (67 - 122)	6.05	147.76

**ENA-78**	16 (13 - 19)	101 (31 - 120)	4.32	45.91

**IL-13**	19 (18 - 23)	26 (19 - 30)	3.25	53.39

**IL-18**	21 (17 - 23)	46 (31 - 54)	3.46	58.95

**IL-6**	34 (24 - 49)	95 (64 - 111)	3.73	94.74

**IL-8**	30 (20 - 38)	66 (36 - 79)	3.83	83.77

**MCP-1**	17 (14 - 20)	56 (35 - 70)	3.95	48.34

**Myeloperoxidase**	36 (31 - 41)	83 (47 - 103)	5.39	100.83

**TIMP-1**	16 (14 - 18)	18 (13 - 21)	5.89	47.23

**VEGF**	19 (14 - 27)	63 (28 - 79)	4.98	54.41

**Median coefficient of variation for all proteins**	32	59	6.05	91.05

**Table 4:** Serum biomarkers found to be reliable and predictive of COPD: P values for linear mixed effects models demonstrating associations between serum biomarker concentrations and lung function and dyspnea.

Serum Biomarker	Post-bronchodilator FEV1	Diffusion capacity (% predicted)	Baseline dyspnea index score
**CRP**	0.13	0.02	0.03

**Myeloperoxidase**	0.03	0.009	0.008

**VEGF**	0.05	0.004	0.003

### Sputum Biomarker Results

Table 5 shows the mean sputum concentrations for the biomarkers retrieved from induced sputum averaged over the 3 patient visits. Of the 22 sputum proteins that were serially assayed, 7 (32%) were found to be significantly associated with COPD. Sputum levels of GROα, TNF RII, IL-6, IL-8, MCP-1, TIMP1, and VEGF were significantly higher in patients with COPD compared to controls. Smoking did not exert a confounding effect; these seven sputum proteins were elevated in both smokers and non-smokers with COPD compared to respective controls. Similarly, adjustment for age and gender did not alter the association between these seven sputum markers and presence of COPD.

**Table 5:** Mean induced sputum protein concentrations averaged over the 3 patient visits:

Sputum Protein	COPD non-smokers	COPD smokers	Control non-smokers	Control smokers	P value COPD vs controls
**Eotaxin2 (pg/mg)**^**#**^	1244.2 (1638.6)	808.9 (349.6)	297.8 (279.1)	1159.5 (1477.7)	0.16

**HGF (ng/mg)**^**#**^	19.2 (15.4)	18.0 (17.0)	7.6 (5.9)	23.3 (20.9)	0.61

**IP-10 (pg/mg)**^**#**^	1015.3 (814.8)	605.8 (393.0)	395.7 (272.9)	480.6 (988.8)	0.94

**KGF (pg/mg)**^**#**^	771.5 (747.7)	881.4 (851.5)	549.9 (516.4)	1490.1 (1256.2)	0.67

**MMP-13 (ng/mg)**^**#**^	29.5 (25.8)	30.9 (23.8)	15.1 (11.7)	45.8 (38.7)	0.99

**TNF-RI (pg/mg)**^**#**^	2459.5 (1548.9)	2635.7 (2280.7)	1390.2 (1241.6)	3671.3 (2806.4)	0.77

**GROα (ng/mg)**^**#**^	54.9 (63.8)	23.3 (18.3)	7.6 (9.6)	15.7 (14.6)	0.007

**TIMP2 (ng/mg)**^**#**^	21.1 (9.2)	18.6 (17.0)	5.8 (5.9)	11.8 (14.4)	0.09

**TNF-RII (pg/mg)**^**#**^	169.1 (113.9)	255.9 (367.0)	46.7 (42.1)	130.4 (129.8)	0.04

α2 macroglobulin (mg/mg)*	0.008 (0.006)	0.004 (0.002)	0.005 (0.003)	0.06 (0.07)	0.09

ENA-78 (ng/mg)*	3.7 (4.1)	1.9 (1.7)	1.4 (1.6)	1.2 (0.8)	0.36

IL-13 (pg/mg)*	21.3 (31.2)	9.3 (7.1)	8.6 (1.9)	5.7 (1.7)	0.92

IL-15 (ng/mg)*	0.17 (0.32)	0.11 (0.10)	0.08 (0.03)	0.04 (0.02)	0.47

IL-18 (pg/mg)*	160.4 (189.8)	118.9 (74.4)	58.4 (39.1)	100.8 (53.2)	0.32

IL-1β (pg/mg)*	142.6 (170.7)	404.3 (701.8)	22.8 (12.3)	53.2 (23.1)	0.06

IL-1RA (ng/mg)*	87.3 (53.1)	107.6 (87.4)	60.7 (36.1)	130.0 (72.1)	0.64

IL-6 (pg/mg)*	156.5 (182.3)	214.4 (194.7)	13.3 (7.9)	100.8 (88.4)	0.00005

IL-8 (ng/mg)*	13.6 (9.0)	32.7 (35.2)	3.5 (3.0)	9.0 (8.8)	0.005

MCP-1 (pg/mg)*	845.2 (1285.2)	692.2 (957.0)	71.3 (54.6)	237.2 (318.6)	0.01

MMP-2 (ng/mg)*	55.3 (37.9)	41.3 (20.4)	60.1 (71.6)	42.3 (20.5)	0.06

TIMP-1 (ng/mg)*	206.6 (71.5)	193.4 (68.9)	75.5 (70.5)	112.3 (83.5)	0.004

VEGF(pg/mg)*	2653.0 (659.6)	2845.7 (1013.8)	1200.4 (706.5)	1733.6 (819.8)	0.006

Values in brackets are the standard deviations of the mean.

P values were assessed using linear mixed effects models adjusted for age and gender (see methods section for details).

All individual sputum protein values are corrected for total sputum protein concentration.

# = Measured using SearchLight multiplex sandwich ELISA

* = Measured using Luminex bead- based suspension array assays

HGF = hepatocyte growth factor, IP-10 = interferon-inducible protein 10, KGF = keratinocyte growth factor, MMP-13 = matrix metalloproteinase 13, TNF-R1 = tumour necrosis factor receptor I, GROα = growth-related oncogene alpha, TIMP-2 = tissue inhibitors of metalloproteinases 2, TNF-RII = tumour necrosis factor receptor 2, ENA-78 = epithelial neutrophil-activating peptide 78, IL = interleukin, IL-1RA = interleukin 1 receptor antagonist, MCP-1 = monocyte chemotactic protein 1, MMP-2 = matrix metalloproteinase 2, TIMP-1 = tissue inhibitors of metalloproteinases 1, VEGF = vascular endothelial growth factor.

Table 6 shows values for within-subject and between-subject mean coefficients of variation for the sputum inflammatory proteins in the COPD cohort. Repeated measurements of these proteins over time showed relatively greater variation in sputum concentrations for most proteins compared to variations in serum protein concentrations. The median intra-subject and inter-subject coefficients of variation were 58% and 79% for sputum proteins respectively, compared to median intra-subject and inter-subject coefficients of variation of 32% and 59% for serum proteins (P = 0.03 for each of the two comparisons). Reference change values for most sputum proteins (Table 6) were high (median reference change value = 163%, range 84% to 238%) suggesting that for most sputum proteins individual measurements in a given patient need to change by >100% from baseline before a statistically significant change in the measurement can be assumed.

**Table 6:** Within-subject and between-subject coefficients of variation, analytical variation, reference change value in COPD induced sputum samples:

Sputum Protein	Within-subject CV (%) (90% CI)	Between-subject CV (%) (90% CI)	Analytical variation (%)	Reference change value (%)
**Eotaxin2**	70 (53 - 95)	88 (50 - 104)	12.1	196.77

**HGF**	58 (44 - 76)	66 (46 - 79)	14.3	165.47

**IP-10**	62 (45 - 82)	54 (32 - 73)	12.2	175.03

**KGF**	49 (33 - 70)	90 (58 - 126)	8.3	137.66

**MMP-13**	54 (36 - 72)	69 (43 - 89)	11.0	152.65

**TNF-RI**	46 (36 - 54)	56 (40 - 65)	8.8	129.73

**GROα**	78 (56 - 100)	89 (52 - 106)	8.2	217.25

**TIMP2**	81 (57- 101)	57 (38 - 77)	8.4	225.57

**TNF-RII**	69 (49 - 89)	104 (65 - 126)	8.6	192.61

**α2 macroglobulin**	57 (33 - 75)	70 (47 - 84)	6.43	158.89

**ENA-78**	61 (49 - 74)	88 (60 - 104)	4.32	169.39

**IL-13**	30 (23 - 37)	101 (32 - 116)	3.25	83.59

**IL-15**	62 (48 - 76)	121 (64- 149)	3.96	172.09

**IL-18**	55 (43 - 66)	63 (32 - 73)	3.46	152.65

**IL-1β**	61 (44 - 77)	160 (113 - 210)	4.58	169.45

**IL-1RA**	58 (46 - 69)	55 (37 - 68)	5.86	161.48

**IL-6**	50 (36 - 68)	99 (59 - 118)	3.73	138.88

**IL-8**	60 (47 - 82)	88 (70 - 106)	3.83	166.54

**MCP-1**	86 (70 - 99)	146 (84 - 182)	3.95	238.47

**MMP-2**	57 (39 - 81)	70 (38 - 82)	3.33	158.16

**TIMP-1**	54 (33- 73)	38 (27 - 46)	5.89	150.47

**VEGF**	42 (32 - 55)	24 (15 - 31)	4.98	117.15

**Median coefficient of variation for all proteins**	58	79	5.88	163.47

For the seven sputum proteins associated with COPD, only IL-6, IL-8, TIMP-1, and VEGF showed acceptable within-subject and between-subject variability over the three patient visits (MCV < 0.6 and BCV < 1.0, respectively).

In the patients with COPD the sputum biomarkers IL-6, IL-8, TIMP-1, and VEGF were significantly and negatively associated with the FEV_1 _(Table 7). All four biomarkers also showed significant association with patient dyspnea and 3 out of 4 were associated with residual volume. Only IL-6 was negatively associated with the diffusion capacity.

Table 8 shows the correlation between sputum biomarker concentrations and total sputum protein concentration As shown in Table 8, most sputum biomarkers with the exception of four, exhibited a strong correlation with total protein.

**Table 7:** Sputum biomarkers found to be reliable and predictive of COPD: P values for linear mixed effects models demonstrating associations between sputum biomarker concentrations and lung function and dyspnea.

Sputum Biomarker	Post-bronchodilator FEV1	Residual Volume (% predicted)	Diffusion capacity (% predicted)	Baseline dyspnea index score
**IL-6**	0.0009	0.007	0.01	0.0007

**IL-8**	0.03	0.24	0.34	0.037

**TIMP-1**	0.004	0.01	0.07	0.009

**VEGF**	0.03	0.01	0.15	0.04

**Table 8:** Correlation between sputum biomarker concentration and total sputum protein concentration.

Sputum Protein	Spearman's correlation coefficient (Rho)	P value
**Eotaxin2**	0.70	0.0005

**HGF**	0.59	0.003

**IP-10**	0.57	0.005

**KGF**	0.37	0.07

**MMP-13**	0.58	0.004

**TNF-RI**	0.64	0.002

**GROα**	0.65	0.001

**TIMP2**	0.69	0.0006

**TNF-RII**	0.67	0.0009

**α2 macroglobulin**	0.51	0.01

**ENA-78**	0.44	0.03

**IL-13**	-0.26	0.19

**IL-15**	-0.18	0.37

**IL-18**	0.54	0.007

**IL-1β**	0.69	0.0006

**IL-1RA**	0.39	0.05

**IL-6**	0.64	0.001

**IL-8**	0.70	0.0005

**MCP-1**	0.69	0.0006

**MMP-2**	0.26	0.20

**TIMP-1**	0.55	0.006

**VEGF**	0.61	0.002

In a posthoc analysis we compared sputum IL-8 levels in patients with COPD who reported chronic bronchitis vs those who did not. Fifteen of the 21 COPD patients reported a history of chronic bronchitis (Table 1). Sputum IL-8 values were significantly higher in COPD patients with chronic bronchits (31.62 ng/mg; SD 35.55) compared to COPD who did not report chronic cough and sputum production (11.20 ng/mg; SD 8.40), P = 0.04.

## Discussion

We found that multiplex immunoassays were useful in identifying potential biomarkers in patients with COPD, and that systemic and sputum biomarkers identified in these patients were associated with clinical variables known to predict disease severity. Importantly, we also showed using repeated measurements over time, that many biomarkers that are apparently associated with COPD exhibit a high degree of intra-subject variability, which renders them relatively unreliable as diagnostic tests if measured on a single occasion.

To establish a criterion for dynamic assessment of biomarkers it is first necessary to define when a difference between two consecutive results indicates a change in a patient's health status. The most widely accepted approach for this purpose is to calculate a reference change value (RCV)^12^. Using serial analytic results from the same patient for a specific biomarker, it is possible to calculate the RCV that defines how large a difference between two consecutive determinations is statistically significant. The RCV encompasses both biological and analytical variation. As seen in Tables 3 and 6 the median RCV values for serum analytes was 91% and for sputum it was 163%. This suggests that for most analytes individual measurements in a given patient have to change by almost 100% (ie. a second measurement must be double, or half, the value of the first measurement) in order to assume that there has been a significant change in the patient's inflammatory status.

Four of 19 serum proteins tested (Eotaxin2, C reactive protein, myeloperoxidase and VEGF) were elevated in patients with COPD compared to control subjects. However of these proteins only CRP, myeloperoxidase and VEGF showed acceptable coefficients of variation to suggest they were sufficiently reliable over repeated measurements, and hence clinically useful as reliable predictors to assess disease state. These were also the only three proteins that showed significant correlations with FEV_1_, dyspnea, and diffusion capacity, suggesting that higher serum levels of these proteins are found in patients with more advanced lung disease.

Our study also found that seven proteins from induced sputum were significantly elevated in COPD subjects compared to controls, however of these seven sputum proteins only four (IL-6, IL-8, TIMP-1, and VEGF) showed acceptable intra and inter-patient reliability. We again found significant correlations of these four proteins with measures of lung function, air-trapping and dyspnea.

It should be noted that sputum biomarker levels were reported normalized for the sputum total protein concentration. We corrected for total sputum protein concentration in order to overcome variability associated with sample dilution of induced sputum. All of our sputum analytes except four showed a significant correlation with total protein. The failure of these four to correlate with total protein concentration suggests dilution alone does not account for the differences seen. However there is also a potential drawback to this method since airway inflammation may increase local production of cytokines as well as protein leakage into the airway from the interstitium. If total protein in the airway is increased due to inflammation, correction of individual biomarkers for total protein concentration will dampen the inflammatory biomarker signal. However we felt that it was important to be conservative and to correct for any variability in dilution of sputum which is likely to be large.

Our study findings are somewhat complementary to those of a recently published study by Sapey and colleagues [13]. These investigators repeatedly measured IL-1beta, TNF alpha, IL-8, myeloperoxidase, LKTB-4 and growth-related oncogene alpha from the spontaneous sputum of 14 patients with COPD. They showed that there was significant intra and inter-patient variability in all of these sputum inflammatory indices which could be reduced by using a 5 day rolling mean of individual patient data points. Results of our study are similar to Sapey's in that we also found significant intra- and inter-patient variability in repeated measurements of sputum inflammatory markers. However our study differs from the Sapey study since we used induced rather than spontaneous sputum. We also evaluated serum biomarkers, and we compared sputum and serum results from COPD patients to control subjects.

Results from our study suggest that variability in serum inflammatory biomarkers is somewhat less than that seen in biomarkers from induced sputum. This finding makes intuitive sense, since the amount of sputum yielded from the lower respiratory tract, and its purulence and relative dilution, can vary in clinically stable subjects from day to day. Correction of results by normalizing for the sputum total protein concentration, such as we did, can partially, but not completely account for variability in sputum yields. In contrast, peripheral blood analysis, which yields a standard amount of serum, is less subject to variability in sampling, and this may explain why inflammatory protein measurements are more reproducible from serum than from induced sputum.

Previous studies have associated serum values of CRP with COPD [3,14]., and CRP has been used as a clinical marker to predict an increased risk of COPD exacerbations [15]. Our study confirmed that serum CRP is significantly elevated in COPD subjects relative to controls. However we also showed similar associations for serum VEGF (vascular endothelial growth factor) and myeloperoxidase. Both these biomarkers are associated with destruction and repair of lung tissue. VEGF is a potent mediator of angiogenesis which appears to be involved in the development of abnormal pulmonary vascular remodeling in COPD [16,17], and myeloperoxidase is a marker of neutrophil activation and inflammation [18,19]. In our study these biomarkers correlated significantly with FEV1, dyspnea, and impairments in diffusion capacity, suggesting that elevation in concentrations of these serum biomarkers may be reflective of ongoing lung destruction, emphysema, and alveolar remodeling.

A previous study by Gompertz and colleagues suggested that the sputum inflammatory markers neutrophil elastase and IL-8 were significantly elevated in COPD patients with alpha-1 antitryspin deficiency and chronic sputum expectoration compared to levels in similar patients who did not chronically expectorate [20]. Our study similarly suggests that patients with a baseline history of chronic bronchitis (defined as chronic cough and sputum production for at least 3 months in the previous 2 years) had higher sputum IL-8 values compared to those COPD patients who did not report chronic cough and sputum and this should be borne in mind when analyzing data from COPD subjects.

There are some limitations to our study. Other sputum inflammatory markers aside from IL-8 may potentially also be higher in the subgroup of COPD patients with chronic bronchitis, however our study did not have adequate patient numbers or power to explore the association of sputum analytes in the chronic bronchitis subgroup in greater detail. We did not perform high-resolution CT scans in our patients to quantitatively describe their degree of emphysema. This would have been useful, in order to try to better phenotype our patients, and in order to relate relative levels of serum biomarkers to CT scan findings of emphysema. Also we did not test for all of the possible proteins that might participate in the complex inflammatory mechanisms that underlie COPD. Other potential limitations relate to potential sources of variability in measurements. Serum and sputum samples were frozen and batched, and a variable duration of freezing, and the freeze-thaw cycle, may introduce variability in measurement values. Two complementary multiplex assays were used, and each platform has its own analytical variation of measurement for each protein. Although we performed spiking experiments for serum proteins, spiking experiments were not performed to measure the same proteins in sputum supernatants. It is possible that the coefficient of variation for analytes measured in serum may not be applicable to that measured in sputum if the biologic fluid affects the assay. Finally, our sample size of 42 subjects was relatively small, and an increase in sample size would be expected to improve the precision around the estimates of mean analyte values in patients with COPD.

## Conclusion

The acceptability of single biomarker measurements can only be interpreted in the context of total variability of the measurements over time. Based on relatively high intra-subject variability and large reference change values for individual biomarkers measurements seen in this study, we would conclude that levels of many serum and sputum biomarkers cannot be reliably ascertained based on single measurements, and that multiple measurements over time can give a more reliable and precise estimate of the inflammatory burden in clinically stable patients.

Our study did identify 3 biomarkers from serum and 4 biomarkers from sputum that were significantly associated with COPD, and with COPD disease severity. Importantly, all of these biomarkers also showed acceptable inter- and intra-subject reliability over repeated measurements. Increasing use of multiplexed immunoassay technology may help to identify new clusters of important biomarkers that may be associated with ongoing lung inflammation of COPD.

## Abbreviations

COPD: chronic obstructive pulmonary disease; TNF alpha: tumor necrosis factor alpha; IP-10: interferon-inducible protein 10; TNF-R1: tumor necrosis factor receptor I; TNF-RII: tumor necrosis factor receptor 2; TIMP-2: tissue inhibitors of metalloproteinases 2; HGF: hepatocyte growth factor; KGF: keratinocyte growth factor; MMP-2: matrix metalloproteinase 2; MMP-13: matrix metalloproteinase 13; GROα: growth-related oncogene alpha; CRP: C-reactive protein; ENA-78: epithelial neutrophil-activating peptide 78; IL: interleukin; MCP-1: monocyte chemotactic protein 1; TIMP-1: tissue inhibitors of metalloproteinases 1; VEGF: vascular endothelial growth factor; IP-10: interferon-inducible protein 10; KGF: keratinocyte growth factor

## Competing interests

SDA has no competing interests.

KLV has no competing interests.

TR has no competing interests.

CZ, ZA, TN and AQ are employees of Roche Pharmaceutical Inc.

## Authors' contributions

SA and KV designed the study and collected the patient data. SA wrote the first draft of the paper.

TR and CZ performed the statistical analysis of the data and edited the paper. ZA, TN, and AQ helped design the study, coordinated assays of the blood and sputum samples, and edited the paper. All authors have read and approved the final manuscript.
